# Translating evidence into practice: adapting TrialGPT for real-world clinical trial eligibility screening

**DOI:** 10.1093/jamia/ocag006

**Published:** 2026-02-04

**Authors:** Mahanazuddin Syed, Muayad Hamidi, Manju Bikkanuri, Nicole Adele Dierschke, Haritha Vardhini Katragadda, Meredith Zozus, Antonio Lucio Teixeira

**Affiliations:** Department of Population Health Sciences, University of Texas Health Science Center at San Antonio, San Antonio, TX 78229, United States; Department of Population Health Sciences, University of Texas Health Science Center at San Antonio, San Antonio, TX 78229, United States; Department of Population Health Sciences, University of Texas Health Science Center at San Antonio, San Antonio, TX 78229, United States; Department of Population Health Sciences, University of Texas Health Science Center at San Antonio, San Antonio, TX 78229, United States; Glenn Biggs Institute for Alzheimer’s & Neurodegenerative Diseases, University of Texas Health Science Center at San Antonio, San Antonio, TX 78229, United States; Department of Population Health Sciences, University of Texas Health Science Center at San Antonio, San Antonio, TX 78229, United States; Glenn Biggs Institute for Alzheimer’s & Neurodegenerative Diseases, University of Texas Health Science Center at San Antonio, San Antonio, TX 78229, United States

**Keywords:** clinical trial eligibility, artificial intelligence, patient screening, electronic health records

## Abstract

**Objectives:**

To evaluate the performance of a locally deployed adaptation of *TrialGPT*, a large language model (LLM) system for identifying trial-eligible patients from unstructured electronic health record (EHR) data.

**Materials and Methods:**

*TrialGPT* was re-engineered for secure, deployment at UT Health San Antonio using a locally hosted LLM. It was optimized for real-world data needs through a longitudinal patient–encounter–note hierarchy mirroring EHR documentation. Performance was evaluated in two stages: (1) benchmarking against an expert-adjudicated gold corpus (*n* = 149) and (2) comparative validation against manual screening (*n* = 55).

**Results:**

Against the expert-adjudicated corpus, the system achieved 81.8% sensitivity, 97.8% specificity, and a positive predictive value of 75.0%. Compared with manual screening, it identified more than twice as many truly eligible patients (81.8% vs 36.4%) while preserving equivalent specificity.

**Conclusion:**

The adapted *TrialGPT* framework operationalizes trial matching, translating EHR data into actionable screening intelligence for efficient, scalable clinical trial recruitment.

## Introduction

Although electronic health records (EHRs) are now widely adopted, patient recruitment for clinical trials remains a major barrier in translational research.^1^^,^^2^ Even after decades of investment in research, participation remains low. For instance, only about 7% of adults with cancer enroll in treatment studies, and fewer than one in five take part in any form of clinical research.^3^ The proportion of people with Alzheimer’s disease enrolled in clinical trials is also low because of multiple logistical and operational barriers, including infrequent referrals, limited access to diagnostics, and inadequate engagement of people from underrepresented groups.^4^^,^^5^

In practice, clinical trial recruitment still relies heavily on manual screening, where study coordinators review patient records and carefully assess each case against detailed trial eligibility criteria.^6^ Structured EHR queries can help narrow the search, but key details such as symptoms, comorbidities, and clinical assessments are often buried in unstructured notes, making the process inherently slow and resource intensive.^7^^,^^8^

Recent advances in artificial intelligence (AI), particularly large language models (LLMs), have opened new possibilities for leveraging free-text clinical data in trial recruitment.^9^ PRISM^10^ and similar frameworks^6^ have demonstrated the potential of LLMs for patient–trial matching, but dependence on proprietary or cloud-hosted models and partial code release limits reproducibility, adds operational burden, and impedes institutional deployment.^11^^,^^12^ In contrast, the open-source *TrialGPT* framework,^13^ introduced in 2024, advanced the field by providing a transparent, well-documented architecture that used LLMs to interpret trial criteria and patient documentation for automated eligibility assessment. However, the original *TrialGPT* focused on a patient-centric use case, matching a single patient, represented by a clinician-provided summary or case description, to potentially relevant trials on *ClinicalTrials.gov* using cloud-based inference. In contrast, our institutional need was trial-centric: enabling study teams to identify potentially eligible patients for a specific study directly from unstructured EHR data. This required processing large volumes of existing clinical narratives at scale rather than manually entered summaries, and generating candidate lists for review instead of evaluating one patient at a time. These fundamental differences necessitated re-engineering *TrialGPT* for institutional deployment, privacy compliance, and scalability.

To address these limitations, we adapted and extended *TrialGPT* for institutional deployment at UT Health San Antonio (UTHSA), transforming it into an operational framework for real-world trial eligibility screening. This study evaluates the adapted *TrialGPT* system for its ability to identify trial-eligible patients from unstructured EHR narratives, comparing its performance with expert adjudication and manual screening within a real-world clinical trial.

## Materials and methods

### Study design and data sources

This study was conducted at UTHSA, where the adapted *TrialGPT* framework was implemented and evaluated for its feasibility and performance for identifying patients eligible for a real-world clinical trial (NCT06585787). The system was applied to a one-year cohort of patients. The evaluation cohort included 149 unique patients with 308 encounters documented in the EHR. For each encounter, the progress note, a comprehensive clinical narrative describing the visit, was used as the sole data source. All notes were processed within a HIPAA-compliant secure environment under institutional review board oversight, with no data transmitted outside institutional boundaries.

### System architecture and workflow

The adapted *TrialGPT* framework builds on the open-source version originally released by the National Cancer Institute (NCI), which links patient documentation to trial criteria through three modules: retrieval, matching, and ranking. While preserving this core architecture, we re-engineered the framework for institutional deployment and integration within real-world clinical research workflows at UTHSA.

To enable operational use in real-world clinical research, the system replaced external API calls with a locally deployed, open-weight large language model (Mistral 7B Instruct^14^) and incorporated a patient–encounter–note hierarchy to represent the longitudinal structure of EHR data. This configuration allows scalable inference over unstructured clinical narratives while ensuring privacy-preserving, reproducible operation entirely within institutional boundaries.

Importantly, the adaptation preserved the original *TrialGPT* retrieval–matching–ranking approach while replacing the proprietary API model with a locally hosted, open-weight LLM. The scoring and aggregation methods were maintained without modification to ensure comparability with the published framework and to enable evaluation in a real-world institutional setting.

At the workflow level, progress notes were extracted from the EHR, standardized to plain text, and linked to corresponding patient and encounter identifiers. Although optimized for trial-centric screening (identifying eligible patients for a specific study), the architecture remains bidirectional, capable of supporting patient-centric matching to recommend appropriate trials for individual patients. All processing occurred within a secure environment powered by four NVIDIA RTX 6000 Ada GPUs (48 GB each), with notes processed individually without chunking since document lengths were within model context limits. Outputs were securely stored within institutional infrastructure, enabling longitudinal analyses, auditability, and integration with ongoing research operations. To promote transparency and reproducibility, the adapted *TrialGPT* framework is openly available at (https://github.com/penad4ut/UTSA-HSC-TrialGPT), with periodic updates reflecting validated enhancements.

### Validation procedure

Validation was comprised of two stages designed to assess system-level accuracy and real-world screening equivalence: (1) evaluating the adapted *TrialGPT* against an expert-adjudicated gold standard, and (2) evaluating the adapted *TrialGPT* against a real-world study coordinator screening patients for the clinical trial. In addition, coordinator feedback on manual screening time was collected to approximate human effort and model relative workflow efficiency.

#### Stage 1—evaluation of TrialGPT against expert-adjudicated gold standard reference (*n* = 149)

The full cohort of 149 patients seen by the specialist provider was independently reviewed by two of three reviewers (two physician-informaticists and one clinical research informaticist with a doctorate in public health) to determine protocol-based eligibility. Discrepancies were adjudicated by the principal investigator (PI) who as the provider for the 149 patients was very familiar with the cases. The resulting decisions formed the Gold Standard Corpus, that is, the expert-adjudicated reference standard. *TrialGPT* was applied to the same cohort, generating relevance, eligibility, and total match scores for each patient. Scores ranged from negative to positive, with values below 0 corresponding to ineligible cases and values approaching 3 indicating strong eligibility alignment. Records with a *TrialGPT* total score ≥ 2 were classified as predicted matches. Model outputs were compared with the Gold Corpus to calculate accuracy, sensitivity, specificity, and positive predictive value (PPV), with 95% Wilson confidence intervals.

#### Stage 2—evaluating the adapted TrialGPT against a real-world study coordinator screening patients (*n* = 55)

**Figure 1.**
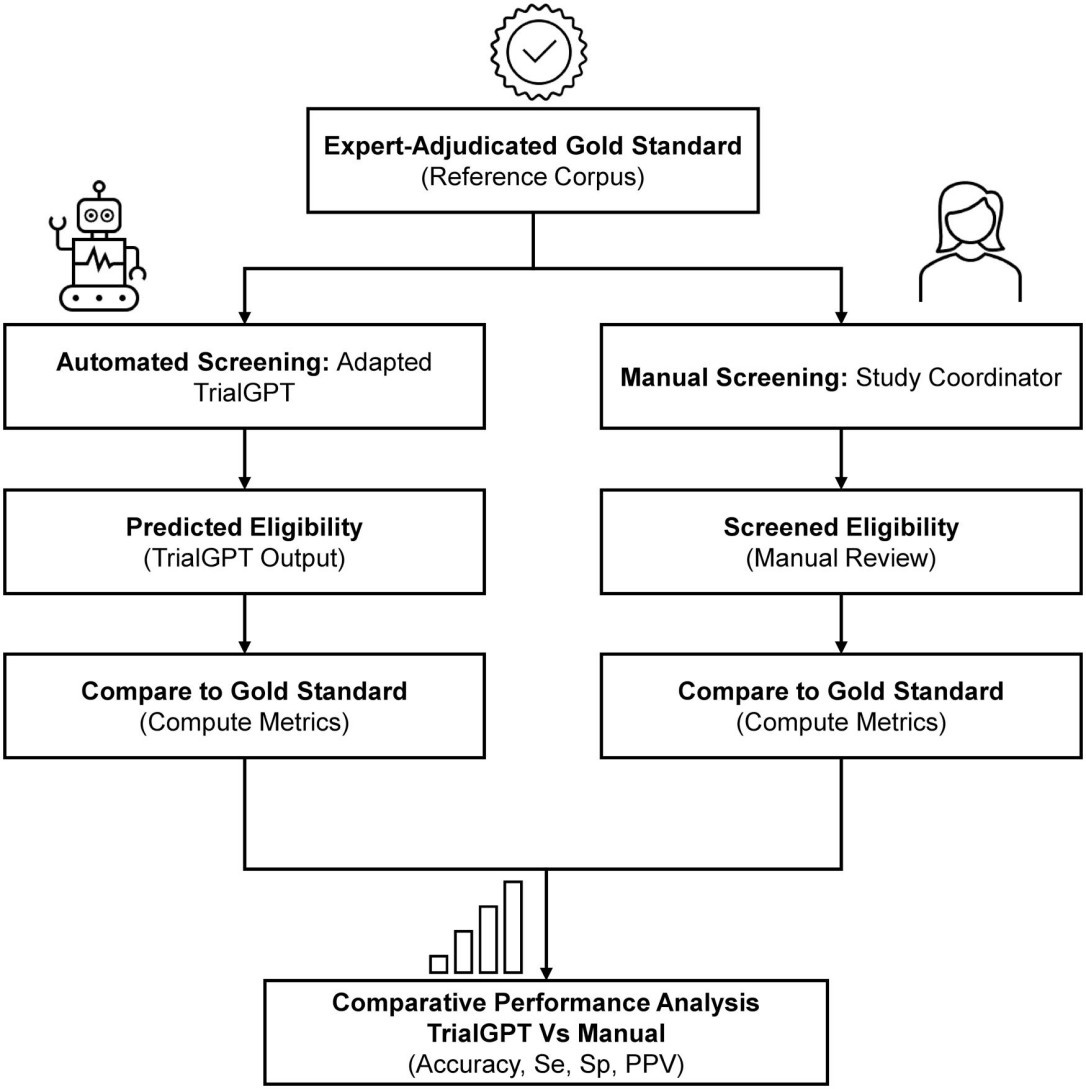
Evaluation design for comparing adapted *TrialGPT* and manual screening against the expert-adjudicated Gold Standard. Each method was evaluated independently, and accuracy, sensitivity (Se), specificity (Sp), and positive predictive value (PPV) were computed relative to the Gold Standard. Comparative analysis of these statistics evaluated the degree of agreement with the Gold Standard and the relative ability of each method to identify trial-eligible patients.

To assess performance against the current manual process for patient screening from narrative clinical documentation, a subset of 55 patients identified by *TrialGPT* as moderate-to-strong potential matches (scores 1-3) was selected. This subset reflected the current practice of querying EHR data to get a list of patients that are as potentially eligible as possible, and the practical constraints of investigator-led recruitment, where clinical staff balance ongoing screening with limited review capacity.^15^ Within this subset, both the *TrialGPT* predictions (≥ 2 = match; ≥ 1 and < 2 = no match) and manual screening determinations by a clinical trial screener were compared independently to the Gold Corpus, using the same reference for both methods. This approach enabled consistent, side-by-side evaluation of automated and manual screening performance under a unified expert standard (Figure 1).

## Results

### Stage 1—evaluation of TrialGPT against expert reference (*n* = 149)

Across the full cohort, the adapted TrialGPT demonstrated high concordance with the Gold Corpus. Of the 149 patients screened, 9 were truly eligible (true positives) for the trial and 135 were correctly identified as ineligible (true negatives), with 3 false positives and 2 false negatives. This corresponded to an accuracy of 96.7% (95% CI 92.4-98.6), sensitivity of 81.8% (95% CI 52.3-94.9), specificity of 97.8% (95% CI 93.8-99.3), and positive predictive value (PPV) of 75.0% (95% CI 46.8-91.1). These findings indicate that TrialGPT achieved high overall accuracy and rarely misclassified ineligible patients as potentially eligible.

### Stage 2—comparative evaluation of automated and manual screening (*n* = 55)

In the focused subset, both *TrialGPT* and manual screening were evaluated against the Gold Corpus. As summarized in Table 1, *TrialGPT* achieved an accuracy of 90.9% (95% CI 80.4-96.1) with markedly higher sensitivity (81.8%) than manual screening (36.4%), while maintaining strong specificity (93.2%) and precision (75.0%). Manual screening showed marginally higher specificity (97.7%) and precision (80.0%), reflecting a more conservative human approach, but this came at the expense of sensitivity. Overall, the re-engineered *TrialGPT* identified a larger proportion of truly eligible patients while preserving comparable accuracy, suggesting its value as a scalable first-pass pre-screening tool in real-world clinical trial workflows.

**Table 1.** Comparative validation of *TrialGPT* and manual screening against the gold corpus (*n* = 55).

Method	**Accuracy** ^a^	**Sensitivity** ^a^	**Specificity** ^a^	**Positive predictive value** ^a^
**TrialGPT (New intervention)**	**90.9 (80.4-96.1)**	**81.8 (52.3-94.9)**	93.2 (81.8-97.7)	75.0 (46.8-91.1)
**Manual screening (Traditional)**	85.5 (73.8-92.4)	36.4 (15.2-64.6)	97.7 (88.2-99.6)	80.0 (37.6-96.4)

a 95% confidence intervals (CIs) calculated using the Wilson method.

### Estimated operational efficiency

**Figure 2.**
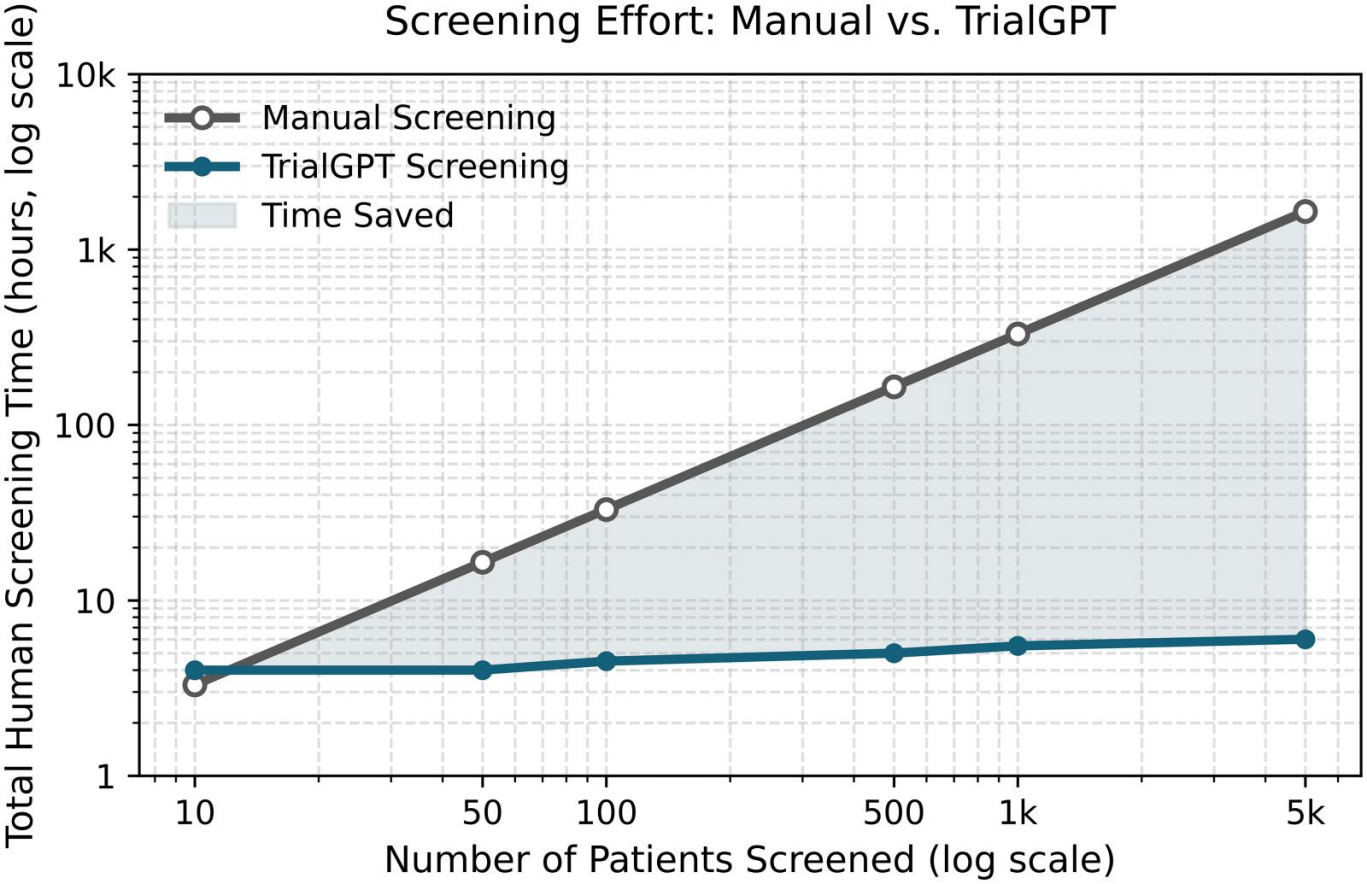
Modeled screening effort for manual and TrialGPT workflows. Both axes use logarithmic scales. Manual effort increases with cohort size, while automated effort remains nearly constant after setup.

In addition to strong performance metrics, automation yielded notable efficiency gains. On average, manual chart review required about 20 minutes of coordinator effort per patient, with complex cases demanding more extensive review across multiple notes and screens. In contrast, the automated workflow involved roughly four hours of one-time human setup, exclusive of unattended model processing. Using these estimates, total human screening time was linearly extrapolated to illustrate scalability beyond the evaluated cohort (*n* = 149). As shown in Figure 2, manual effort scales proportionally with cohort size, whereas human effort for the automated workflow remains largely constant, highlighting its scalability for larger studies.

Collectively, these findings demonstrate that automated LLM-based pre-screening can substantially expand identification of eligible patients without increasing false positives, thereby augmenting manual workflows while maintaining high overall accuracy. Building on these findings, we next examined the time and effort savings achievable through automation using study coordinator-reported estimates.

## Discussion

This study demonstrates that a locally adapted version of *TrialGPT* can accurately identify trial-eligible patients from unstructured EHR narratives while eliminating reliance on proprietary API models and maintaining privacy, reproducibility, and institutional compliance. Compared with manual screening, the system achieved higher sensitivity while maintaining comparable specificity, positioning it as a practical, institution-ready tool to enhance the efficiency and consistency of manual patient prescreening. These findings highlight the feasibility of embedding LLM–based screening within existing clinical research workflows, transforming free-text documentation into actionable trial recruitment intelligence. This evaluation focused on system performance and feasibility within existing institutional workflows.

Replacing commercial API calls with a locally deployed model ensured privacy, reproducibility, and cost efficiency.^13^ Unlike the research prototype, which could not be used institutionally under HIPAA restrictions, this re-engineered version demonstrates that LLM–based trial matching can be securely integrated and scaled locally within institutional infrastructure. Compared with commercial solutions that often require dedicated computing environments and extensive vendor onboarding,^11^^,^^12^ this streamlined configuration provides a feasible path for institutions to adopt AI-driven screening within existing governance processes. Collectively, these advances bridge the gap between experimental AI frameworks and operational deployment in real-world clinical research.^16–18^

When benchmarked against the expert-adjudicated gold corpus, the adapted system closely aligned with expert review, achieving high sensitivity and specificity. In comparative validation, it identified substantially more eligible patients than manual screening while maintaining comparable specificity. Manual reviewers, often constrained by time and uncertainty,^15^ tended to exclude borderline cases, whereas *TrialGPT* applied consistent eligibility reasoning across all encounters, mitigating human variability and uncovering missed opportunities for enrollment.

Analysis of misclassified cases provided insight into model behavior. The two false negatives were borderline cases with total scores just below the predefined eligibility threshold (≈1.7), indicating that minor threshold adjustments or adaptive calibration could capture such near-miss cases without compromising specificity. Among false positives, two patients with Lewy Body disease were misidentified as Alzheimer’s disease related psychosis, and one patient with a recent cardiac exacerbation was incorrectly included despite a safety exclusion in the protocol. These examples highlight how inconsistently worded eligibility criteria, particularly those involving comorbidities or exclusion conditions, can create uncertainty for both human reviewers and AI systems. Clearer, standardized eligibility language in future trial designs could strengthen interpretability, enable more reliable technology-assisted screening, and support responsible AI adoption in clinical research.

Architecturally, the adapted system introduces a patient–encounter–note hierarchy, scoring each encounter independently and aggregating results at the patient level. This structure mirrors how eligibility is determined in practice while maintaining scalability within institutional data systems. Although effective operationally, it does not yet capture temporal relationships between encounters, an avenue for continued future development.

Limitations include the single-site, single-trial study for a very specific clinical condition used for this evaluation. This may limit generalizability across institutions and domains. The general-purpose Mistral model also lacks domain-specific fine-tuning for eligibility reasoning. Future work will focus on testing the application across multiple studies, therapeutic areas, and open-weight LLM configurations, coupled with progressive integration into clinical trial management system and EHR workflows to enable real-time operational deployment. Planned enhancements include continuous domain-specific adaptation, integration of structured EHR data, and Health Level Seven (HL7) Fast Healthcare Interoperability Resources (FHIR) enabled interoperability, along with clinician-in-the-loop dashboards to support real-time, transparent trial screening.

## Conclusion

This work demonstrates how translational informatics can operationalize AI frameworks within real-world research infrastructure. The adapted *TrialGPT* framework shows how research-grade AI tools can be refined to operate securely, reproducibly, and at scale within clinical research environments. By transforming unstructured EHR narratives into actionable screening intelligence, this work advances the translation of AI from experimental models to practical, trustworthy systems that strengthen clinical research infrastructure.

## Data Availability

The adapted TrialGPT framework code is publicly available at https://github.com/penad4ut/UTSA-HSC-TrialGPT. The clinical data used for evaluation were derived from institutional electronic health records and cannot be shared publicly due to patient privacy and HIPAA restrictions.
